# “It Is Like That, We Didn't Understand Each Other”: Exploring the Influence of Patient-Provider Interactions on Prevention of Mother-To-Child Transmission of HIV Service Use in Rural Tanzania

**DOI:** 10.1371/journal.pone.0106325

**Published:** 2014-09-02

**Authors:** Annabelle Gourlay, Alison Wringe, Isolde Birdthistle, Gerry Mshana, Denna Michael, Mark Urassa

**Affiliations:** 1 Faculty of Epidemiology and Population Health, London School of Hygiene and Tropical Medicine, London, United Kingdom; 2 National Institute for Medical Research, Mwanza, Tanzania; Duke University Medical Center, United States of America

## Abstract

Interactions between patients and service providers frequently influence uptake of prevention of mother-to-child transmission (PMTCT) HIV services in sub-Saharan Africa, but this process has not been examined in depth. This study explores how patient-provider relations influence PMTCT service use in four government facilities in Kisesa, Tanzania. Qualitative data were collected in 2012 through participatory group activities with community members (3 male, 3 female groups), in-depth interviews with 21 women who delivered recently (16 HIV-positive), 9 health providers, and observations in antenatal clinics. Data were transcribed, translated into English and analysed with NVIVO9 using an adapted theoretical model of patient-centred care. Three themes emerged: decision-making processes, trust, and features of care. There were few examples of shared decision-making, with a power imbalance in favour of providers, although they offered substantial psycho-social support. Unclear communication by providers, and patients not asking questions, resulted in missed services. Omission of pre-HIV test counselling was often noted, influencing women's ability to opt-out of HIV testing. Trust in providers was limited by confidentiality concerns, and some HIV-positive women were anxious about referrals to other facilities after establishing trust in their original provider. Good care was recounted by some women, but many (HIV-positive and negative) described disrespectful staff including discrimination of HIV-positive patients and scolding, particularly during delivery; exacerbated by lack of materials (gloves, sheets) and associated costs, which frustrated staff. Experienced or anticipated negative staff behaviour influenced adherence to subsequent PMTCT components. Findings revealed a pivotal role for patient-provider relations in PMTCT service use. Disrespectful treatment and lack of informed consent for HIV testing require urgent attention by PMTCT programme managers. Strategies should address staff behaviour, emphasizing ethical standards and communication, and empower patients to seek information about available services. Optimising provider-patient relations can improve uptake of maternal health services more broadly, and ART adherence.

## Introduction

Interactions between health care providers and their patients are widely recognised to play an important role in determining the uptake of health services [1]. The providers' role in influencing medication adherence, health-seeking behaviour, and satisfaction with health services has been documented [2], [3], with studies on patient-provider interactions focussed predominantly on the developed world primary care setting and treatment of chronic conditions such as cancer [4].

Interest in patient-provider interactions in the context of HIV care has grown as HIV infection has been transformed from an acute to a chronic condition with the advent of antiretroviral therapy (ART). Several studies from the developed world have reported quantitative associations between measures of patient-provider relations and patient satisfaction with HIV care, and ART or appointment adherence [5]–[8]. Qualitative research on this topic is less common, but is helpful in revealing *how* interactions between patients and providers influence HIV service use, offering insight for health-system strengthening [9], [10].

Evidence of links between patient-provider interactions and uptake of HIV services in the developing world has been presented in recent systematic reviews of qualitative and quantitative literature investigating barriers and facilitating factors to ART access and adherence [11], [12]. One review, focussing on uptake of antiretroviral (ARV) drugs in the context of prevention of mother-to-child transmission (PMTCT) HIV services in sub-Saharan Africa, highlighted the importance of interactions between pregnant HIV-positive patients and health workers [12]. However, despite the potential influence of these interactions on PMTCT outcomes, no studies have examined their consequences in detail.

**Figure 1:**
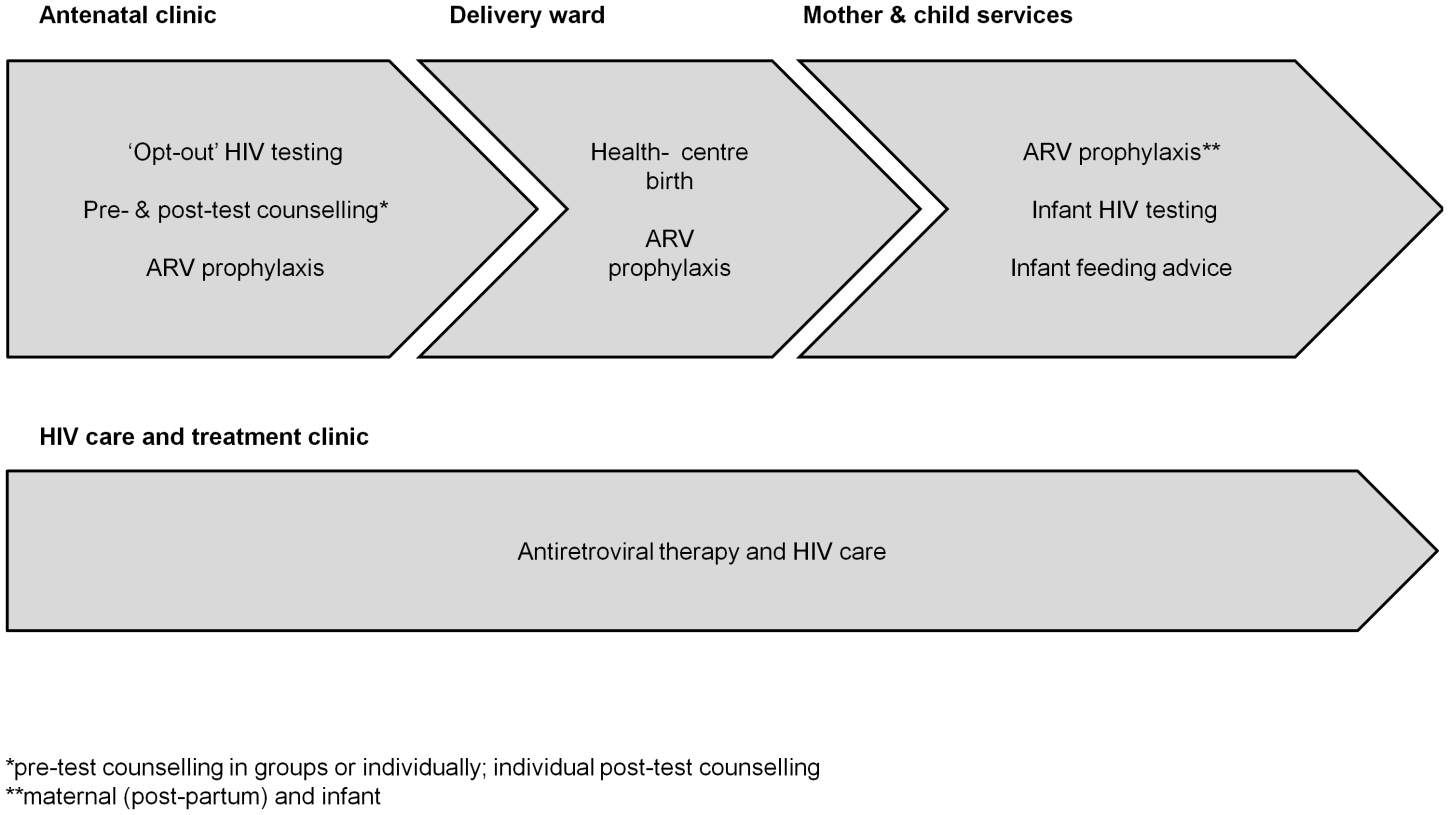
Cascade of PMTCT services by clinic location.

The PMTCT programme comprises multiple potential contact points with providers within a cascade of services, including antenatal clinic (ANC) visits, ‘opt-out’ HIV testing with pre- and post-test counselling, ARV drugs for those diagnosed HIV-positive, facility-based deliveries, infant ARV prophylaxis and infant HIV testing (figure 1). As HIV-positive women are encouraged to initiate prophylaxis or ART early in pregnancy to prevent vertical HIV transmission, and continue treatment for life for their own health [13], PMTCT services are shifting towards a chronic care model, with the need for repeated clinic visits for drug refills and adherence monitoring. As such, patient-provider interactions within the PMTCT programme are increasing in frequency and importance.

In Tanzania, government HIV treatment programmes were introduced nationally in 2004, with ART provided free of charge to HIV-positive individuals meeting eligibility criteria [14]. Maternal and child health (MCH) services are also provided (theoretically) without cost. However, despite decentralisation efforts, HIV care and treatment clinics (CTC) remain largely restricted to hospitals and health centres in urbanised areas, due to a lack of funding and personnel. PMTCT services, first implemented nationally in 2000, have since been extended to include HIV testing and ARV prophylaxis provision in health centres and rural dispensaries, although stock-outs are frequent [15]. In Tanzania, as in many other African countries, low PMTCT service use remains a major obstacle to global targets for elimination of new paediatric HIV infections and reduction of maternal mortality [16].

We therefore sought to explore the nature of patient-provider interactions within PMTCT service provision in rural Tanzania, and ways in which these interactions influence the uptake of PMTCT services, with the aim of providing recommendations for optimising patient-provider relations and PMTCT uptake.

### Theoretical perspectives

Patient–provider interactions have been conceptualised with a range of theoretical models, evolving over time from traditional paternalistic or biomedical models of care, towards a more ‘patient-centred’ approach. Balint first introduced the theory of patient-centred medicine in the 1950s [17]; more recent definitions include “seeing the illness through the patient's eyes” [18]. The approach is thus distinct from paternalistic or biomedical models, in which physicians make decisions in the best interest of the patient with minimal patient involvement and a focus on the patient's biomedical problems.

A literature review by Mead and Bower identified five dimensions of patient-centred care: bio-psychosocial perspectives, ‘patient-as-person’, ‘doctor-as-person’, a ‘therapeutic alliance’, and ‘sharing power and responsibility’ [19] (table 1). Shared decision-making, a feature of shared power, has been described as the ‘crux of patient-centred care’ [20], and was considered a key indicator in an evaluation of interventions to promote patient-centred care [3]. An element of shared decision-making and an essential component of patient-centred care is effective communication and information sharing [21]. Researchers have also discussed how patient-centred approaches can alleviate disparities in care based on ethnicity, race and socioeconomic status [22], [23]. Other components of patient-centred care frameworks include continuity of care [22] and structural factors such as the availability of resources and time [24].

**Table 1:** Key elements of patient-centred care conceived by Mead and Bower.

**Dimensions of patient-centred care**	**Description or examples**
Bio-psychosocial perspective	Covering social and psychological issues, not only medical aspects of care
Patient-as-person	Differences in individuals' experience of illness
Doctor-as-person	Personal qualities of the doctor and self-awareness
Therapeutic alliance	Including personal bond between doctor and patient; doctors being caring and empathetic
Sharing power and responsibility	Including shared-decision making
**Influencing factors**	**Examples**
Doctor factors	Personality, gender, age
Patient factors	Attitudes or expectations, age, knowledge
‘Shapers’	Cultural norms
Professional context influences	Performance incentives, government policy
Consultation-level influences	Workload pressures, time limitation

The philosophy of patient-centred care is now widely adopted in medical practice in the developed world, for example in general practice and nursing [25], reflecting a growing recognition of its importance in delivering high quality care services. Frameworks for patient-centred care have therefore been extended to include measurable outcomes such as patient satisfaction with care and quality of life [22], [25]. A Cochrane review concluded there was some evidence that interventions promoting patient-centred care (e.g. training health workers) were associated with increased patient satisfaction [3]. There are fewer examples of the application of the patient-centred care model in the developing world, although authors have recently advocated for a shift towards patient-centred approaches in infectious disease and reproductive health services in lower income countries [26].

## Methods

### Study setting and design

Kisesa is a relatively poor rural area in north-western Tanzania with a low GDP per capita of <500 US dollars (USD). Most residents earn a living from subsistence farming or small businesses selling local produce. There are four government health facilities in Kisesa: three rural dispensaries in remote villages and one health centre in the trading centre. The district and regional referral hospitals are ≥20 km away. PMTCT services have been operating (intermittently) in all four facilities since 2009, although comprehensive HIV services are only offered in the health centre (since 2008). Regular rounds of demographic and HIV serological surveillance have been conducted in Kisesa, among a population of approximately 30,000, since 1994.

A variety of qualitative methods - participatory learning and action (PLA) group activities with mothers and fathers, in-depth interviews (IDI) with HIV-positive and HIV-negative women as well as health providers, and observations of procedures and patient-provider interactions in ANC and MCH clinics - were used in a broader study investigating barriers to PMTCT service use in Kisesa in 2012. PLAs extend more commonly used focus group discussion methods to include further participation from participants, such as role-playing or creating charts or maps, promoting a two-way exchange of knowledge and information between researchers and participants. PLA methods had been useful previously in this setting, to investigate barriers to accessing other HIV services, engaging community members and capturing prevalent community-wide beliefs which complement individual perspectives from IDIs [27]. PLA activities were also designed to generate a locally relevant vignette (short story) about a hypothetical pregnant woman diagnosed with HIV to aid discussion during the IDIs given the sensitive topic area, and limit reporting bias [28].

The study was designed to maximise learning from different methodologies and perspectives, including those of HIV-positive women, to explore direct experiences with PMTCT services, and HIV-negative women, for more general experiences of MCH services, and the wider community and health service providers.

### Sampling and recruitment

PLA activities were conducted with 3 male and 3 female groups from different residence areas (remote (RE), roadside (RD), trading centre (TC)). Participants were randomly selected from a sampling frame, constructed using demographic and HIV sero-surveillance datasets, of 3102 community members aged 15–60 who had ≥1 child, for experiences or views on MCH services. A few HIV-positive women were purposively included in each female PLA group using an approach based on the ‘seeded’ focus group [29]. Fieldworkers were unaware of the HIV status of any individuals. Overall, 30% of 105 individuals visited were not found, and 2% refused to participate. Each group included 8–12 participants. Demographics of PLA participants are summarised in table 2. Male participants were slightly older than female participants, with an average age of 42 years compared to 36 years respectively.

**Table 2:** Characteristics of PLA group participants (3 male groups and 3 female groups each with 8–12 participants, N = 61 participants in total).

	Female PLA groups	Male PLA groups
Characteristic	Number (%) of participants (n = 30)	Number (%) of participants (n = 31)
**Age**		
19–29	7 (23)	5 (16)
30–39	13 (43)	8 (26)
40–49	7 (23)	12 (39)
50–59	2 (7)	6 (19)
unknown	1 (3)	0
Mean (range)	36 (19–54)	42 (24–59)
**Area of residence**		
Remote rural	12 (40)	10 (32)
Roadside	10 (33)	9 (29)
Trading centre	8 (27)	12 (39)
**HIV status**		
HIV-positive	8 (27)	0
HIV-negative	21 (70)	15 (48)
Unknown	1 (3)	16 (52)

IDIs were conducted with 21 women who had been pregnant or given birth since 2009. Ten HIV-positive women were recruited purposively by nurses from each clinic, while eleven women (5 HIV-negative, 6 HIV-positive) were recruited from the community via PLAs, ensuring a mix of women with and without experience of ANC or PMTCT services. Women were discreetly invited for interview by fieldworkers after the PLAs, using a coded list. Fieldworkers were unaware of participants' HIV status, unless participants voluntarily disclosed their status during interviews. Of 8 HIV-positive women invited, 6 accepted. All 5 HIV-negative women accepted. Recruitment continued until data saturation was reached, based on preliminary analyses. Characteristics of women participating in the IDIs are presented in table 3. Participants were aged between 20 and 47 years, with an average age of 34 years. Roughly half lived in more remote rural villages, the other half residing in the trading centre or roadside villages. Among the subset of women recruited from the community for whom data on education was available, most were educated to primary level (as were the majority of women in this community).

**Table 3:** Characteristics of female community members participating in IDIs (N = 21).

Characteristic	Number (%) of participants (n = 21)
**Recruitment method**	
From community (PLA)	11 (52)
By clinic nurse	10 (48)
**Age**	
20–29	4 (19)
30–39	11 (52)
40+	4 (19)
unknown	2 (10)
Mean (range)	34 (20–47)
**Area of residence**	
Remote rural	10 (48)
Roadside	5 (24)
Trading centre	6 (29)
**HIV status**	
HIV-positive	16 (76)
HIV-negative	5 (24)
**Year of most recent delivery**	
2009	3 (14)
2010	5 (24)
2011	11 (52)
2012	2 (10)
**Education**	
None	1 (5)
Primary	10 (48)
Secondary +	0
Unknown	10 (48)

Six health workers and three officials were interviewed; recruited purposively to include most of the providers directly involved in delivering PMTCT services in the study area, and to provide perspectives from different facility levels and cadres. Five MCH nurses, a doctor at the CTC, and coordinators at the district and referral hospital were selected and agreed to participate. The health workers interviewed were mostly female (5 out of 6), while two out of three health officials were male. Half of the health workers practised in smaller dispensaries and half worked in the health centre.

### Data collection

PLAs were facilitated in Kiswahili by fieldworkers following a written protocol of activities, including group discussions, role-plays about a woman attempting to access PMTCT services (participants created their own characters and stories), brainstorming of barriers along the PMTCT cascade, and ranking of barriers.

Trained interviewers conducted IDIs lasting 1–3 hours in Kiswahili. The semi-structured discussion guide included a vignette [28], personal experiences of recent pregnancies, and overall perceptions of services received. Interviewers probed for MCH services accessed, health worker conduct, privacy, counselling offered, and clarity of medical advice. Twelve of sixteen known HIV-positive interviewees voluntarily disclosed their status and discussed PMTCT services.

A fieldworker interviewed health workers in Kiswahili, while the principal investigator interviewed health officials in English. Discussions included challenges with delivering PMTCT services, and perceived difficulties for patients.

Observations were also conducted in communal areas of Kisesa health centre ANC and MCH clinic by the principal investigator and one fieldworker. Structured observation sheets included prompts for patient-provider interactions, privacy, procedures, patient volume and waiting times.

### Data analysis

Photographs of physical outputs and detailed notes were taken during PLAs. After each PLA or interview, debriefing sessions were held to discuss emerging themes. PLAs and interviews were digitally audio-recorded, following consent from participants, transcribed verbatim, then translated into English.

Analysis was conducted in NVIVO9, guided by a framework approach [30]. Mead and Bower's patient-centred care framework (table 1) was used to organise the initial code-frame, which also incorporated aspects of patient-centred care emphasized by other researchers [22]–[24]. The code-frame was refined after applying it to the first few transcripts; adding, sub-dividing and combining codes. Coding was also done inductively to capture new concepts, and participants' own terminology was documented through in-vivo codes. Codes were then grouped into overarching themes.

The resulting code-frame was applied to all transcripts (‘indexing’) by identifying content that described dialogue or other interactions between patients and providers, and the extent to which interactions were patient-centred (e.g. discussion of non-medical matters, question-asking by patients, statements suggesting empathy, included in most coding schemes for analysing verbal behaviour and patient-centred care [19]). The manner in which patients described being treated and questioned by providers (and vice versa), and links to outcomes (e.g. adherence to PMTCT components) were also scrutinized. Perceptual data, for example generated when discussing the vignette, was also analysed to reinforce perspectives from personal experiences. Charts were created using MS Excel to compare data across and within cases.

The principal investigator read and coded all translated documents. A sub-sample of transcripts was double-coded (by AW) using the same initial code-frame. Each researcher's revised code-frame was then compared and discussed to ensure all emerging concepts were captured.

### Ethics statement

Ethical approval was granted by the London School of Hygiene and Tropical Medicine, Tanzanian Lake Zone and Medical Research Coordinating Committee ethics review boards.

All participants were informed about the study. Before commencing the PLAs, verbal consent was recorded from all participants using a digital audio recorder. It is important to offer verbal consent in this setting due to the expected low level of literacy of some of the participants and the fact that people are not used to signing their name as a form of consent. For some HIV-infected women, the existence of a signed consent might be perceived as an unjustified threat to the subject's confidentiality. All IDI participants were given the option of written or verbal consent, for the same reasons outlined. No participants recruited for interview or PLAs were minors aged <18, therefore consent was obtained from all individuals in person, rather than from next of kin, guardians or caretakers. These procedures for obtaining participant consent were approved by the ethical review boards.

Recruitment procedures, using the seeded focus group method and including HIV-negative women, were designed specifically to maximise confidentiality for HIV-positive women. PLA participants were advised that the discussion was confidential, but they were not expected to share personal information. Compensation was provided for travel expenses (5000 Tanzanian shillings, 3 USD). Participant anonymity was maintained by using fictitious or generalised names, and labelling recordings, transcripts and quotations with codes (e.g. ‘health worker#1’).

## Results

**Figure 2:**
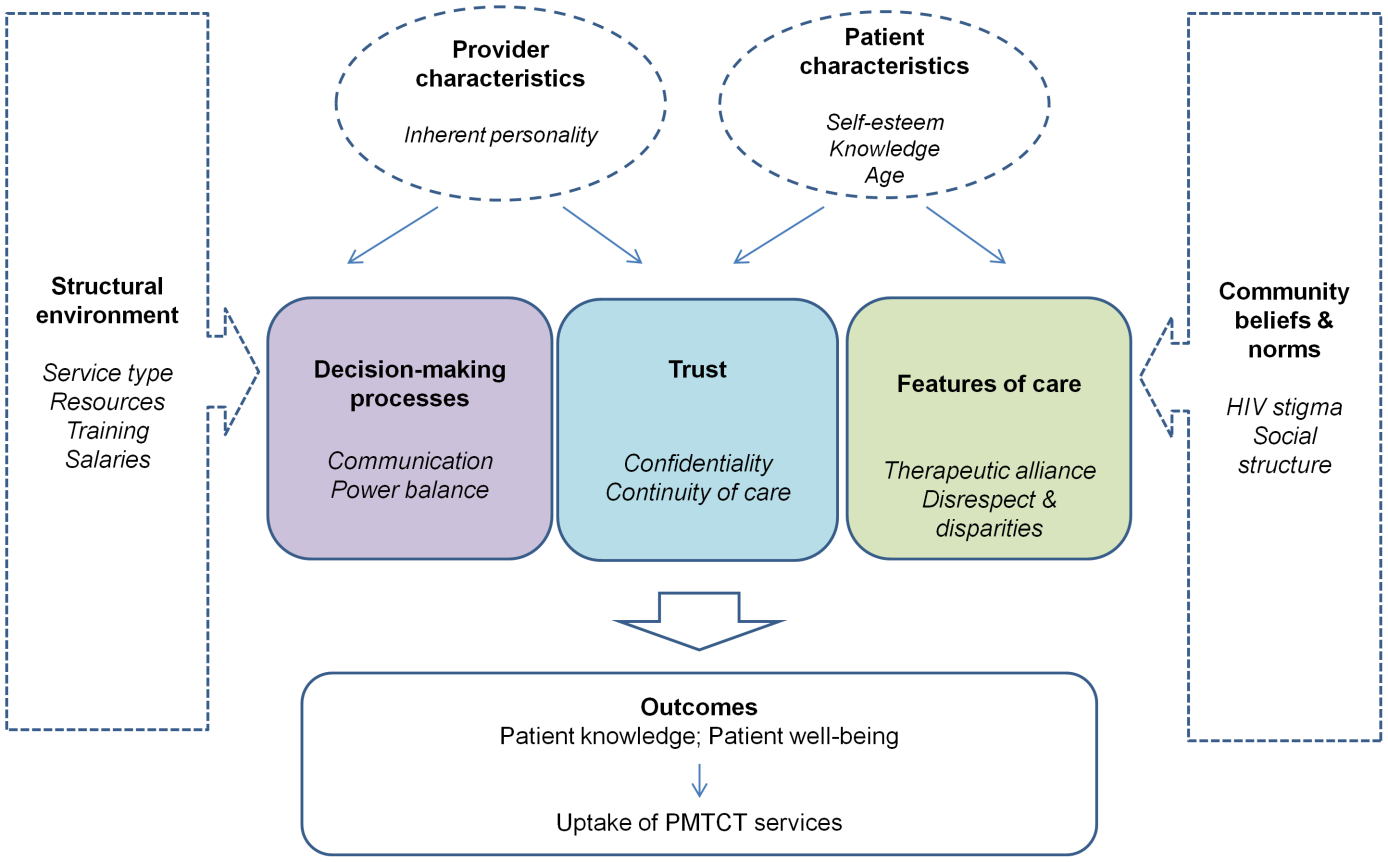
Conceptual framework for the analysis of patient-provider interactions in Tanzania.

Three themes emerged relating to patient-centred care: decision-making processes, trust, and features of care. Decision-making processes encompassed the sub-themes of communication, including psycho-social support, and power balance; clear and non-threatening communication by providers, and willingness of patients to engage in discussion and clarify information, both necessary for shared decision-making. Trust in providers was related to continuity of care with a single provider. Features of care included the therapeutic alliance and disparities in care. We also identified provider and patient characteristics that influence the process and nature of interactions. Interactions are shaped by the structural environment and community perceptions and norms. Patient-provider interactions affected patient knowledge, well-being, and ultimately access to and retention in PMTCT services. Building on the patient-centred care model, our adapted framework is presented in figure 2, and was used to guide synthesis of our findings.

### Decision-making processes

#### Communication

All IDIs with health workers and a few with HIV-positive women revealed examples of providers offering psycho-social support. Nurses described assisting HIV-positive patients to come to terms with their diagnosis, in one case helping a patient to adhere to the PMTCT programme, take ARV drugs and deliver an HIV-negative baby.

*I became very sad when I saw my results, but I gave myself courage…I just consoled myself because of that counselling (IDI, HIV-positive women#16)*

Health workers showed intricate knowledge of their patients' personal lives including relationship challenges and family circumstances. The goal of testing male partners for HIV sparked further discussions about psycho-social issues, including disclosure strategies and personal plans for their involvement in testing and support during pregnancy.

*The woman was getting the treatments here…she was beaten by her husband when she said ‘I'm stopping breastfeeding the child’. We talked with her- she said I don't want to infect the child (Health worker#4)*

Some health workers explained that psycho-social counselling could galvanise support from relatives, thus improving a woman's chances of accessing services such as delivery.

Effective communication, through the transfer of knowledge to the patient or psycho-social support, influenced the uptake of PMTCT services. A few HIV-positive women, who described advice as encouraging or helpful, or apparently understood instructions for taking medicines, said this motivated them to take the drugs or attend the clinic. Simply being given advice by the provider, regardless of quality, was often linked to perceived or actual use of PMTCT services, such as ARV drugs and infant testing. However, receiving and understanding advice did not always improve PMTCT service adherence, in light of more influential factors such as economic constraints, distance from facilities or partnership issues. Some providers explained how despite their efforts to provide counselling, patients could still refuse to take prophylaxis.

*You counselled her from the first months up to the last, but she didn't like to use [the drugs] at all, someone can completely reject those medicines (Health worker#3)*

A minority of women interviewed received basic explanations for HIV testing during pregnancy, and a nurse reported providing education about HIV transmission at ANC. However, the majority of women reported not receiving any formal pre-test counselling, or explanations of the testing process. This concurred with observations in the health centre, where formal group counselling sessions were rarely observed. A few women said there were sometimes group classes before HIV testing, but that they had arrived late, missed the counselling and were just tested (without individual counselling).

*I: Before they test, don't they give you any counselling?*

*R: They just tell me ‘sit there so we can check your health’ (IDI, HIV-negative woman#5)*

Poor communication about the HIV testing process and the right to opt-out appeared to have an impact on patient understanding, since many women believed they could not opt-out and had often received no counselling or explanations about testing. Some female PLA participants also articulated there was no possibility of declining the test. A common belief was not receiving further pregnancy services if they did not test.

*You won't be provided service if you are not tested…you can't refuse, you must be tested.*

*(IDI, HIV-positive woman#12)*

*When I went to test I remember the nurse didn't tell me anything....others are saying that when you go there to test, the nurse will first give you counselling – she will ask you ‘are you ready’, but I remember she didn't ask me. I didn't get any counselling. (IDI, HIV-negative woman#6)*

Health workers generally inferred that patients could decline the HIV test, though one male PLA participant suggested providers communicated in a coercive style.

*It [HIV test] is voluntary, but there is a language which is used to convince her*

*(Male PLA participant, TC)*

Other examples of unclear communication, resulting in confusion or lack of understanding for patients, included a woman who did not understand what was happening when she was admitted to hospital for a labour complication because doctors were speaking in English. In other cases, scant explanations were given about the reasons for performing medical examinations, for issuing advice regarding facility-based deliveries or breastfeeding, or for prescribing drugs including ARVs.

*I don't know what those tablets help; you are just given and you swallow them*

*(IDI, HIV-negative woman#1)*

However, unclear communication did not necessarily impede PMTCT service use. For example, some women reported taking drugs despite confusion over their purpose. Lack of pre-test counselling and misunderstandings about the right to opt out of the HIV test may, controversially, have improved uptake of testing. Nonetheless, thorough explanations about testing had positive implications: a few interviewees received some explanation which motivated them to test. The perception that HIV testing is compulsory and associated fear of results could also deter women from attending ANC, as discussed by several PLA participants.

#### Power balance

The best example of shared decision-making, patient autonomy and power sharing was the case of one woman who negotiated with the nurse to receive the labour dose of antiretroviral prophylaxis in advance, which she consequently took, despite delivering at home at night.

*They asked me when I was 8 months pregnant – ‘where do you live, do you have a person at home who can help you?’ I said I do not, and home is far away…maybe I can fail to get there [hospital delivery ward]…That is why I explained openly to the nurses. They gave me those medicines [ARVs] (HIV-positive woman#7)*

Patients and providers occasionally described discussing personal plans for what time of day to take ARV medication, or infant feeding options. All health workers and a few women mentioned asking or being asked questions, particularly in discussions around ARV drug adherence or disclosure plans. An HIV-positive woman said ‘*you can even remind the nurse*’ to dispense ARV drugs. Some nurses also mentioned receiving reminders from patients about ARVs, particularly when they were busy and had forgotten, clearly facilitating drug adherence. One provider noted that when HIV test kits were unavailable, patients occasionally asked why they were not tested.

However, overall there were very few examples of patients asking questions, resulting in missed opportunities to receive vital services or drugs. One HIV-positive woman specifically stated it was not her place to question the provider.

*They didn't tell me [infant's HIV test results]…the problem is that they [health workers] are the people who have to say’ (IDI, HIV-positive woman#17)*

In several cases, women said they had not questioned or clarified with the provider, despite not receiving information (e.g. test results) nor fully understanding (e.g. about the purpose of prescribed drugs). A combination of poor provider communication and patient subservience led to one woman not receiving her positive HIV test result until after delivery, and no ARV prophylaxis.

*R: The nurse didn't tell me if I was supposed to wait or come back tomorrow [for HIV test results]…when I finished [testing] I left.*

*I: So why didn't you ask about your results?*

*R: It is like that, we didn't understand each other (HIV-positive woman#18)*

A male PLA participant said some women were unable to express themselves in the presence of a doctor, relating this to lack of education.

Terminology used by interviewees and community members to describe providers reflected the attributed power: terms such as ‘experts’, ‘specialists’ and ‘experienced’ were frequently used. Information from providers was referred to as ‘conditions’, ‘directions’, and ‘instructions’, while phrases like being ‘ordered’ by health workers, or ‘violating the conditions’ were also common.

This appeared to have both negative and positive consequences for PMTCT uptake. Fear of being reprimanded for ‘violating the conditions’ was commonly perceived to hinder clinic access, yet the provider's power and their ‘instructions’ also facilitated adherence to advice. Many patients said they had followed or accepted advice because they were told to, or imagined this would enable adherence to PMTCT services.

*I told her [nurse] that I am ready to know my status, because you are the one who made the diagnosis and found I am infected, I will not deny the results*

*(HIV-positive woman#7)*

*I: But what do you think will make her [vignette character] swallow the drugs?*

*R: It is only the directions; they [nurses] tell her that you must follow these directions*

*(HIV-positive woman#13)*

### Trust

#### Confidentiality

One participant explicitly expressed trust in her provider, while lack of trust in health workers emerged more frequently, for example not trusting providers to keep a woman's HIV status confidential, particularly because they were ‘local people’. However, confidentiality breaches were not documented. All women said their consultations were private, where discussed. Several nurses mentioned keeping their patients' HIV status secret, or reiterating this to patients. One commented “I don't think there is a worker giving secrets out”, while another acknowledged the potential implications for patient retention: “when you let out that secret that patient will not come again”. Lack of trust in health workers due to incompetency (e.g. apparent loss of test results, or scepticism regarding HIV test results) was also mentioned, and contributed to patients defaulting from HIV care.

#### Continuity of care

Trust in providers was also related to continuity of care and familiarity with staff. A few nurses described how pregnant HIV-positive women were anxious about being referred to other providers (e.g. due to lack of ARVs or HIV test kits), after having established trust in their original provider.

*If she starts being given the service by you, it will be you only…, she doesn't want another worker to know that thing [her status]. Now if you send her to another place, she sees as if I'm going to start there afresh (Health worker#2)*

Lack of continuity in care also occurred when the usual nurse was not on duty, because nurses were expected to cover a range of services owing to staff shortages, leading to drop-outs or missed PMTCT components.

*One [patient] can need only you to serve her. She can find you not present, and doesn't trust the other who is present, so she turns back*

*(Health worker#6)*

*I tested that day, I didn't get the results, I went back home, I came the next month but I didn't find the nurse who tested me. I returned for the third time then I was given the results: my result was positive.*

*(IDI, HIV-positive woman#18, did not receive ARV prophylaxis during pregnancy)*

### Features of care

#### Therapeutic alliance

Women occasionally portrayed helpful, caring, polite, kind, compassionate or forgiving staff, or a personal bond with their provider.

*The nurse herself was very kind, she received me well…she just looks for me, she asks me (IDI, HIV-positive woman#21)*

Interviews with some nurses also revealed empathetic conduct, for example paying for patients' delivery materials with their own money (confirmed by two women), and expressions of sympathy regarding their patients' personal circumstances.

*You even go to buy [gloves] if you have some money, your own money, because you love the job (Health worker#6)*

Participants often described being ‘well received’, ‘well attended’, welcomed or helped, or expressed satisfaction; sometimes specified as reasons for returning to PMTCT facilities. However, this was usually explained by the receipt of services, such as pregnancy checks or drugs, lack of scolding, or the health workers not having any ‘problems’.

*She [the nurse] was of help to me… because she was giving me all the services*

*(IDI, HIV-positive woman#13)*

*I: How did the health workers attend you?*

*R: Just good*

*I: When you say good what do you mean?*

*R: Because they didn't scold me*

*(IDI, HIV-positive woman#7)*

#### Disrespect and disparities in care

Many HIV-positive and HIV-negative women gave accounts of disrespectful and demeaning staff behaviour including scolding, insulting language, not being provided services, nor being treated with dignity, particularly during delivery. Lack of materials (e.g. gloves, sheets, basins) at facilities, and the need for women to purchase and bring these items for delivery, exacerbated the situation and fuelled corruption.

*I went to the health centre and at the time of delivery…they asked me for the rubber sheet and I told them I didn't have anything. I had a lot of problems getting her [the nurse], I had to deliver on the floor*

*(IDI, HIV-negative woman#8)*

*Every nurse has his/her price… they totalled the whole cost: it was 17,000 Tanzanian shillings [10 USD]....the injection, gloves, everything. Some nurses will order you to pay 5,000 only [3 USD], others 6,000 [3.6 USD], and others reach up to 20,000 shillings [12 USD]*

*(Female PLA participant, TC)*

HIV test kit and drug stock-outs caused further tensions, including blame on both sides, while health workers also expressed disappointment at not being able to test pregnant women for HIV.

*But now there are no medicines, the woman will just ask you ‘how is it that my child stops taking the medicines’? (Health worker#1)*

Inappropriate health worker behaviour related to stock-outs of materials was ranked among the greatest barriers to using PMTCT services by two female PLA groups (TC, RD). Women and men from remote villages recognised these issues, but they were ranked below factors such as distance from services and economic hardship. Health officials also identified staff behaviour as a major challenge for pregnant women participating in the PMTCT programme.

*What is important is the staff at the MCH clinic. When someone is harsh or rude, it can hamper your service - some [staff] are very rude, and do not use good language, so the woman is fearful…she may return home… so the language and behaviour of those providers is very, very, very important (Health official#1)*

Several HIV-positive interviewees experienced discrimination by health workers or anticipated stigmatising attitudes. Discrimination was manifest in verbal insults, not receiving services, or being told by nurses to wash their own clothes after delivery.

*That nurse…sometimes she would tell you…‘you are suffering from AIDS, you will also give birth to a baby with diseases, you will suffer’*

*(IDI, HIV-positive woman#21)*

*You are supposed to wash them [delivery clothes] yourselves…so that you may not infect the person who is attending you*

*(IDI, HIV-positive woman#13)*

Discrimination at health facilities towards pregnant women in general, particularly young or old women, and was also described occasionally.

*I am too old [38], we are very much insulted at the hospital (IDI, HIV-positive woman#7)*

*After that she touches you, then she will tell you…to raise your dress, she can't touch your body because she feels you are dirty…This is one way of stigmatising someone irrespective of whether she is HIV infected or not. In short…pregnant women are just really being harassed (Female PLA participant, TC)*

Pregnant women were also scolded when going to ANC too early or too late (e.g. 3 months, or 7–8 months gestation respectively).

Perceptions of poor behaviour by staff, expressed in responses to the vignette, or PLA role-plays and discussions, were also frequently described and mirrored individuals' own experiences. Experienced negative staff behaviour during pregnancy, or fears of scolding, (e.g. for failing to take ARVs, or home-births) influenced adherence to subsequent steps of the PMTCT programme, such as facility-based deliveries, returning for infant prophylaxis, or infant HIV testing. For example, one HIV-positive woman became fearful, discontinued ART and did not take her child for an HIV test after having blood drawn by a ‘rude’ nurse.

*She is afraid of going to the clinic because she was not going there during her pregnancy, and if you were not going to the clinic it will be difficult to go to the hospital to deliver…They will ask for your clinic card… it will show that she was not attending…they will scold you (IDI, HIV-positive woman#10)*

#### Provider characteristics

The provider's inherent character was proposed by PLA and IDI participants as a reason for poor behaviour and differences in conduct, though some felt health system factors (e.g. low salaries, lack of resources, poor training) were largely to blame.

*Insults are just about someone's character. Money can't change someone who has that habit…the habit is something inborn (Male PLA participant, TC)*

Provider character and care quality were also linked to service type, with a few HIV-positive women noting more respectful care in HIV services compared to general MCH services.

*They [HIV-nurses] are not rude like our nurses who render ordinary services (HIV-positive woman#19)*

## Discussion

This is the first qualitative study to explore in depth the ways in which patient-provider interactions influence the uptake of PMTCT HIV services in Africa, providing specific evidence to guide approaches to optimise PMTCT service provision.

Overall, there were few examples of shared decision-making. Instead, breakdowns in communication were common, and the balance of power leaned strongly towards the provider. Women generally perceived their social status below that of the provider. This has been described in another Tanzanian nursing study [31], and reflects wider gender and cultural norms, respect for those in positions of authority and the inherent and assumed power structure, although power differentials between patients and health service providers are not uncommon in developed world settings [32]. Our observations may also relate to the socio-demographic profile of women in our study who were from a relatively poor rural area and had a fairly limited education. The few examples of patients questioning or negotiating with providers, thereby averting missed opportunities, suggest that empowering women may be an important strategy to improve PMTCT service use. Women have increasingly been involved in the AIDS activist movement in South Africa, lobbying for improved access to ART and a national PMTCT programme [33]. Links between empowerment of HIV-positive women and enhanced patient-provider relationships have also been suggested by research in the USA [34]. Another American study demonstrated the intermediary role of ancillary services (e.g. treatment advocacy groups) in facilitating patient-provider relationships by, indirectly, assisting HIV-positive patients to develop self-advocacy skills and providing a discussion forum for clarification of medical advice, or directly, by communicating on behalf of patients and accompanying them in medical consultations [35]. In our setting, home-based care workers or support groups may offer potential alternatives to this approach. However, empowerment will inevitably take time, and may not always be desired, as researchers in similar settings have documented that patients appreciate assertiveness [31], [36]. Interestingly, we found that issuing instructions in an authoritative manner sometimes *positively* influenced PMTCT service use. Nonetheless, providers should distinguish giving clear advice and exerting superiority, which may frighten women from returning if they fail to comply with instructions.

Knowledge transfer to the patient appeared to be a pathway through which effective communication might lead to uptake of PMTCT services. Communication effectiveness was quite clearly linked to knowledge transfer, although the link between knowledge acquisition and PMTCT service uptake was less predictable. Women sometimes followed advice without apparently understanding details or reasoning, potentially reflecting the power imbalance. Conversely, other more influential factors (e.g. partnership issues) could override the provision or understanding of advice and ultimately determine adherence to the PMTCT programme, suggesting that high quality counselling and strong patient-provider relationships alone may not suffice. Unclear communication about the HIV testing process and lack of pre HIV-test counselling evidently compromised women's understanding, resulting in misconceptions about the possibility of opting out and, controversially, greater uptake of HIV testing (although this may not necessarily translate into adherence to subsequent PMTCT services). Such results may be a consequence of staff being overburdened; an issue raised by providers in our study, reflecting the difficulties of operating the service under such conditions [37]. Alternatively, health workers may believe they know what is best for their patients, exploiting their assumed superiority, and that minimal explanations may ultimately improve health outcomes for women by increasing testing rates. However, it is imperative that in the quest to achieve high coverage of ARVs for PMTCT, the HIV testing consent process and women's rights are not jeopardised. This calls for clearer guidance on ethical procedures for administering HIV tests at ANC, and attention to the ethics of care within nursing courses. It should be made clear to pregnant women that a decision to opt-out of HIV testing will not affect their eligibility to receive further routine antenatal services. Practices that account for women arriving at ANC at different times should also be implemented, ensuring multiple group pre-test counselling sessions are offered, or individual pre-test counselling and consent for latecomers. Investments in staffing may help to ensure ethical standards are maintained by reducing time pressures resulting from staff shortages.

Amidst a principally paternalistic approach, the detailed knowledge health workers had of patients' personal situations was striking, and reflected an important aspect of patient-centred care. The social implications of an HIV diagnosis and holistic components of HIV programmes, such as HIV testing of male partners at ANC and involvement of relatives in ART adherence training, will naturally trigger discussions about relationship or family circumstances. Although there was little evidence for the direct benefit of such discussions on PMTCT service uptake, this may be mediated through enhanced family support. Support from partners and relatives was an important facilitating factor to uptake of PMTCT services in similar settings [12] and in our broader study findings [37]. The intimacy of some patient-provider relationships, uncommon in most medical contexts, may also partly reflect the rural study setting with low tier health facilities and moderate patient volumes, providing the opportunity to become familiar with the same patients. This dynamic is also likely to echo the local cultural context, as it has been noted previously with nurses in Tanzania [31].

Concerns over lack of provider confidentiality appeared unfounded but are likely to reflect prevailing beliefs and HIV stigma in the community. Tackling HIV stigma at a community level is therefore important, and may also help to shift prejudicial health worker attitudes and practices. Referrals to other facilities disrupted established patient-provider relationships, exacerbated lack of trust, led to anxiety and ultimately drop-outs. Strengthening the PMTCT programme at the primary care level through sufficient stock of HIV test kits and ARV drugs, and continued decentralisation of HIV care services, are therefore likely to facilitate patient-provider interactions and patient retention. Decentralisation, and integration of HIV and ANC services, will be critical to the programme's success as changing global policies, advising all women diagnosed HIV-positive during pregnancy to initiate lifelong ART [13], are implemented. Promoting respectful care, identified as a central component of patients' trust in South African health workers, may also improve trust in providers [38]. Enhancing trust in providers also has the potential to improve ART adherence [8].

Accounts and fears of disrespectful care by health workers were widespread, affecting HIV-positive and negative women, and were particularly shocking at the time of delivery. Disrespectful treatment including scolding, insulting remarks and deliberate non-attendance of deliveries, as well as discrimination of HIV-positive women, have previously been reported in African PMTCT, delivery and primary care services [38], [39], including Tanzania [40]. Disrespectful behaviour was a major deterrent to further participation in PMTCT services, and also requires urgent attention on moral grounds by PMTCT programme managers and policy makers. Addressing this issue also has the potential to improve uptake of maternal health services more broadly, particularly skilled delivery attendance. Strategies to tackle poor health worker behaviour might include enhanced education on patient rights and ethics of care in clinical training courses, accountability for misconduct, incentives for good conduct, and thorough on-job supervision with feedback. Tanzanian primary care workers desired feedback after supervisory visits from district-level staff [41], while the need for more training and feedback was also highlighted by providers in our broader study [37]. Investing in and ensuring appropriate distribution of resources, particularly essential but inexpensive materials for delivery such as gloves, should also be a priority. Our findings suggest it is not only the lack of materials and associated costs that deter women from accessing delivery services, but the resulting tensions between patients and providers. Comments about better care in HIV services, compared to maternal health services more generally, potentially reflect the extra investments made in HIV services. However, if PMTCT HIV services are to be successfully integrated within maternal health services, investments are also needed in the latter. Systemic issues that may exacerbate poor behaviour such as staff shortages, low salaries and lack of incentives, are inherently complicated and challenging to address, but improvements in these areas could reap dividends in improving the uptake of PMTCT services.

The patient-centred care framework was a useful starting point to guide our analysis. Most dimensions of Mead and Bower's model were identified, although other elements of patient-centred care including disparities and continuity in care, the structural backdrop and community-based factors, were also important in shaping patient-provider interactions in this setting. ‘Doctor-as-person’ and ‘patient-as-person’ were not distinguished in our data, potentially reflecting difficulties in measuring these dimensions [19], or the context, in which illness is also seen and experienced through relatives and significant others. We also extended the original framework to include PMTCT outcomes, and intermediary pathways. Our conceptual framework therefore provides a contribution to the evaluation of patient-provider relationships and healthcare delivery, particularly within developing world HIV programmes. While we used this framework as a lens to explore patient-provider interactions, we did not specifically set out to evaluate the extent to which medical interactions were patient-centred, nor presumed patient-centred care to be the ideal model for clinical care in our setting. Vaga et al. specifically challenged the expectations regarding approaches to caring in different socio-cultural contexts such as Tanzania [31]. Nonetheless our findings document a largely paternalistic style of medical care, interspersed with elements of a patient-centred approach.

The range of qualitative techniques and respondent types were strengths of this study, enabling a synthesis of findings from different methodologies and perspectives to enhance validity. Community surveillance datasets facilitated recruitment of HIV-positive women for IDIs who were not necessarily enrolled in PMTCT care, improving validity of findings, and maximising confidentiality. The vignette was designed to minimise social desirability bias and uncover difficulties in using PMTCT services. Nonetheless, self-reported drug adherence and understanding of instructions could not be validated. Health workers are also more likely to report positive interactions with patients. Recall of details relating to pregnancies over a year before fieldwork may also be limited in accuracy. The presence of the (European) principal investigator may have influenced participants' behaviour and interpretation of results, although Tanzanian fieldworkers and researchers were involved in discussions of results.

## Conclusions

The patient-provider relationship plays a pivotal role in PMTCT service use. Addressing the ethical issues surrounding informed consent for HIV testing at ANC, and disrespectful treatment of pregnant women, must be priorities for PMTCT programme managers and policy makers. Strategies should focus on improving staff behaviour, with emphasis on the ethics of care and communication, while empowering women to seek information about essential services. Optimising provider-patient relations can improve uptake of maternal health services regardless of HIV status, and ART adherence beyond PMTCT. Strengthening the capacity of health services, in particular overcoming the equipment shortages that underlie some negative patient-provider interactions, is likely to be very challenging in this setting, but could substantially improve access to and retention in PMTCT and MCH programmes.
